# Field Epidemiology and Public Health Microbiology training: capturing the alumni perspectives of the training’s impact

**DOI:** 10.2807/1560-7917.ES.2023.28.36.2300388

**Published:** 2023-09-07

**Authors:** Justine Schaeffer, Charlotte Christiane Hammer, Iro Evlampidou, Laura Bubba, Zsofia Igloi, Timothée Dub, Annika Wendland, Jane Whelan, Stine Nielsen, Amrish Baidjoe, Alma Tostmann, on behalf of the EPIET Alumni Network Boards 2017–2023

**Affiliations:** 1 EPIET Alumni Network Advisory Board, Saint Maurice, France; 2 Department of Veterinary Medicine, University of Cambridge, Cambridge, United Kingdom; 3 Médecins Sans Frontières, Operational Centre Brussels, Brussels, Belgium; 4 Médecins sans Frontières, Luxembourg Operational Research, Luxembourg; 5 Department of Health Security, Finnish Institute for Health and Welfare, Helsinki, Finland; 6 London School of Hygiene and Tropical Medicine, London, United Kingdom; 7 Epismart, Amsterdam, the Netherlands; 8 Department of Medical Microbiology, Radboud Centre for Infectious Diseases, Nijmegen, the Netherlands

**Keywords:** career mobility, professional education, public health, microbiology, epidemiology, surveys and questionnaires

## Abstract

We present the findings from the European Programme for Intervention Epidemiology Training (EPIET) Alumni Network (EAN) Member Survey conducted in October to December 2021. The EAN consists of field epidemiologists (EPIET) and public health microbiologists (European Public Health Microbiology Training Programme (EUPHEM)) who stay connected after their 2-year fellowship. This active alumni network provides opportunities for career development, mentorship, knowledge exchange and sharing of best practices for community members, affiliated professionals and public health organisations in Europe. Overall, 281 of 732 members participated in the survey. Of the 192 European fellowship alumni respondents, 173 (90%) indicated that skills and competencies acquired during their fellowship improved performance in their role compared with their abilities before the fellowship. Reported skills and competencies that could be further strengthened included data management/analysis, communication, mathematical modelling and leadership/team management. The EAN Member Survey provides valuable feedback to the EAN, as well as the fellowship programme offices at the European Centre for Disease Prevention and Control (ECDC) and affiliated field epidemiology programmes. The COVID-19 pandemic was a stark reminder of how essential cross-border collaborations are for continued European health security. Maintaining and increasing the professional, well-trained workforce remains crucial for optimal response to infectious diseases and protection of public health.

## Background

Serious threats to health, such as infectious diseases, do not respect borders. They do, however, require a streamlined cross-border response. The European Programme for Intervention Epidemiology Training (EPIET) was created in 1995 with the objective to train public health professionals from different (public) health disciplines, as well as different cultures and linguistic backgrounds across Europe, to become field epidemiologists [1]. The goal was to establish common methodologies and a common epidemiological language facilitating harmonised approaches and a highly collaborative international workforce. The European Public Health Microbiology Training Programme (EUPHEM) was initiated in 2008 to strengthen collaboration across different microbiological disciplines and between microbiologists and epidemiologists [2]. In 2016, EPIET and EUPHEM became the ‘European Centre for Disease Prevention and Control (ECDC) Fellowship Programme’, which is focused on prevention and control of infectious disease threats, in accordance with the mandate of ECDC [3,4].

Fellows can follow the European Union (EU)-track, where they are trained in an EU country other than their country of citizenship or, since 2011, the Member State (MS)-track, where they are trained in their country of citizenship [1]. The fellowship programmes are distinguished from classical academic programmes by uniting a solid common theoretical background with training fellows in the setting of day-to-day practice, i.e. ‘the learning-by-doing’ approach [5]. After a 2-year fellowship comprising a comprehensive curriculum, the participants in the training programmes receive their diploma. In this Perspective, we present a summary of the results of membership survey that the Advisory Board of the EPIET Alumni Network (EAN) conducted in 2021.

## Stronger together: the EPIET Alumni Network

The EAN (https://epietalumni.net) provides a community for field epidemiologists and public health microbiologists to stay connected after completing their fellowship, and forms a largely European cross-border professional network. It was established as an independent association in 2000. The EAN members are alumni from EPIET, EUPHEM and ‘EPIET-associated programmes’, including the German Postgraduate Training in Applied Epidemiology [6], and other European national Field Epidemiology Training Programmes (FETPs) such as the United Kingdom (UK)-FETP [7]. The EAN also welcomes ‘external’ members who meet similar objectives to the EAN and can provide relevant expertise to the network [8].

Despite the close bonds formed during the fellowship and the obvious professional benefits, field epidemiology alumni networks such as the EAN are rare. Other well-known and established FETP alumni networks are the United States (US) Centers for Disease Control and Prevention (CDC) Epidemic Intelligence Service alumni network [9] and the global networking platform TEPHI-Connect (https://tephiconnect.org), hosted by the Training Programmes in Epidemiology and Public Health Network (TEPHINET) [10]. Of these, TEPHI-Connect has the greatest geographical reach, bringing together graduates from across all FETPs globally.

The EAN provides opportunities for career development, mentorship, knowledge exchange and sharing of best practices to its members and beyond. The EAN is an active and engaged network, with an annual calendar of scientific training modules, seminars, career sessions for fellows and alumni, and social networking events across Europe. Communication within the network occurs via weekly EAN email bulletins, on the website with a public and a ‘members’ area, and via various social media and communication platforms.

Combining professional exchanges and the informal character of the network cultivates professional trust across borders and institutions. This facilitates easier and more rapid exchange of formal and informal knowledge and information with the proven potential to strengthen essential collaboration during large public health emergencies, such as during the 2014–16 Ebola outbreak in West Africa, the 2020–23 COVID-19 pandemic and the 2022–23 mpox (formerly monkeypox) outbreak response.

### Training programme assessment by EPIET Alumni Network members: 2021 survey

A regular assessment of the ‘time-place-person’ characteristics of the EAN member base is important. Quantifying the diversity of the network, roles and post-fellowship positions, as well as possible changes in competencies and needs over time, provides valuable feedback to the fellowship programme management, to EU policymakers that have means to make additional investments in such programmes, and to the EAN Advisory Board.

Since the last EAN Member Survey in 2013 [11], the ECDC Fellowship Programme has evolved: many EUPHEM alumni have graduated and became part of the EAN, there have been an increasing number of MS-track fellows and the number of fellows per cohort has also increased (data not shown), resulting in a continuously expanding alumni network.

The EAN Advisory Board conducted a follow-up membership survey in 2021, and between September and December 2021, an online questionnaire and follow-up reminders were distributed to all EAN members via email, the weekly EAN bulletin, social media and other platforms, e.g. WhatsApp, Discord. Data were collected on demographics, professional background, current role or position, perceived benefits of the fellowship and the EAN, and involvement in the COVID-19 response. 

### Characteristics of survey respondents

All 732 EAN members were invited and 281 participated (response rate: 38%). The response rate was highest among most recently graduated alumni: 46% were 2014–21 (121/265) alumni who joined the programme after the previous member survey, 38% were 2008–13 (76/202) alumni which includes the first EUPHEM cohorts, and 25% were 1995–07 alumni (38/150). The response rate was highest among EUPHEM EU-track (57%, 30/53), followed by EPIET EU-track (38%, 113/298), EPIET MS-track (36%, 33/91) and EUPHEM MS-track (27%, 8/30).

The first alumni of the EPIET fellowship completed their training 25 years ago, in 1997. Despite this, over a third of our membership base engaged in this survey, confirming the continued commitment of alumni to this network. Even though responses may be overrepresented among EAN members who are active in the network and/or work in public health.

The median age of respondents was 42 years (range: 25–66), 194 (69%) were female, 83 (30%) were male and 4 (1%) did not answer this question. Of the respondents, 146 (52%) were fellows or alumni of EPIET, 38 (14%) of EUPHEM, 67 (24%) of one of the associated FETP-programmes, and 30 respondents (11%) were external members (see Table). Many alumni respondents currently work in national public health institutes (41%), at the World Health Organization (10%) or at sub-national public health entities (9%). The current main areas of work are communication and advice to authorities (23%), data analysis (19%), research (9%) and communication and advice to the public (8%). The main area of work was more often ‘communication and advice to authorities’ among ECDC-MS track alumni (48%) compared with ECDC EU-track alumni (23%).

**Table:** Characteristics of respondents of the EPIET Alumni Network Member Survey, October–December 2021 (n = 281)

Characteristics	All respondents (n = 281)		ECDC EU-track (n = 143)		ECDC MS-track (n = 41)	
	n	%	n	%	n	%
Programme						
EPIET EU-track	113	40.2	113	79.0	NA	
EPIET MS-track	33	11.7	NA		33	80.5
EUPHEM EU-track	30	10.7	30	21.0	NA	
EUPHEM MS-track	8	2.8	NA		8	19.5
PAE (German FETP)	43	15.3	NA			
UK FETP	17	6.0				
Other European FETP	6	2.1				
Non-European FETP	1	0.4				
None	22	7.8				
Missing	8	2.8				
Current status						
EPIET or EUPHEM fellow	37	13.2	19	13.3	10	24.4
Alumnus	244	86.8	124	86.7	31	75.6
Current job: type of organisation^a^	(n = 211)		(n = 122)		(n = 31)	
National public health institute	86	40.8	35	28.7	22	71
World Health Organization	20	9.5	19	15.6	0	0
Sub-national public health institute	19	9.0	8	6.6	5	16.1
Hospital or practice	15	7.1	8	6.6	2	6.5
ECDC	14	6.6	10	8.2	1	3.2
Academia	13	6.2	8	6.6	0	0
Other private sector	11	5.2	11	9.0	0	0
NGO	7	3.3	6	4.9	0	0
Self-employed	5	2.4	3	2.5	1	3.2
Pharmaceutical company	1	0.5	1	0.8	0	0
Other international public health organisation	4	1.9	4	3.3	0	0
Other	16	7.6	9	7.4	0	0
Current job: main area of work^a^	(n = 210)		(n = 121)		(n = 31)	
Communication and advice to authorities	57	27.1	28	23.1	15	48.4
Data analysis	47	22.4	30	24.8	6	19.4
Research	22	10.5	11	9.1	0	0
Communication and advice to the public	19	9.0	12	9.9	4	12.9
Team management	9	4.3	4	3.3	1	3.2
Data management	8	3.8	6	5.0	2	6.5
Routine diagnostics	6	2.9	3	2.5	1	3.2
Clinical work	5	2.4	0	0	2	6.5
Surveillance	4	1.9	4	3.3	0	0
Project management	3	1.4	3	2.5	0	0
Development of diagnostic tests	2	1	2	1.7	0	0
Modelling	2	1	1	0.8	0	0
Teaching and capacity building	2	1	2	1.7	0	0
Outbreak investigation	1	0.5	1	0.8	0	0
Other	23	11	14	11.6	0	0
Current job: disease area^a^	(n = 210)		(n = 121)		(n = 31)	
Respiratory diseases	39	18.6	28	23.1	2	6.5
Generalist	39	18.6	19	15.7	6	19.4
Other	39	18.6	17	14	12	38.7
Hospital-acquired infection	26	12.4	10	8.3	5	16.1
Vaccine preventable diseases	22	10.5	21	17.4	1	3.2
Zoonotic infections	21	10	12	9.9	0	0
Food and waterborne	5	2.4	3	2.5	0	0
Non communicable diseases	5	2.4	2	1.7	1	3.2
Sexually transmitted diseases	5	2.4	1	0.8	1	3.2
Vector borne diseases	5	2.4	4	3.3	3	9.7
Blood borne diseases	4	1.9	4	3.3	0	0
Where was your first job after the fellowship?^a,b^ n (%) 'yes'	(n = 211)		(n = 143)		(n = 41)	
Country of citizenship	109	51.7	49	34.3	28	68.3
Country of fellowship	71	33.6	36	25.2	13	31.7
Other country in Europe	24	11.4	16	11.2	1	2.4
Outside of Europe	27	12.8	22	15.4	0	0
Remote	5	2.4	4	2.8	0	0
Where have you mostly worked after the fellowship?^a,b^	(n = 211)		(n = 143)		(n = 41)	
Country of citizenship	143	67.8	74	51.7	29	70.7
Country of fellowship	79	37.4	45	31.5	14	34.1
Other country in Europe	77	36.5	59	41.3	3	7.3

ECDC: European Centre for Disease Control and Prevention; EPIET: European Programme for Intervention Epidemiology Training; EU: European Union ; EUPHEM: European Public Health Microbiology Training Programme; FETP: Field Epidemiology Training Program; MS: Member State; NA: not available; NGO: non-governmental organisation; PAE: Postgraduate Training for Applied Epidemiology (German FETP); UK: United Kingdom.

**^a^** Current fellows excluded (n = 37).

^b^ More than one answer was possible.

The majority of alumni responding to this survey work in in-country public health entities, fulfilling the main objective of the fellowship to strengthen the EU workforce. The high proportion of respondents working with the World Health Organization (WHO) may be a biased outcome, as this could be largely due to the COVID-19 pandemic (i.e. the time of the survey) during which WHO had hired many short-term consultants.

### Home away from home

**Figure 1:**
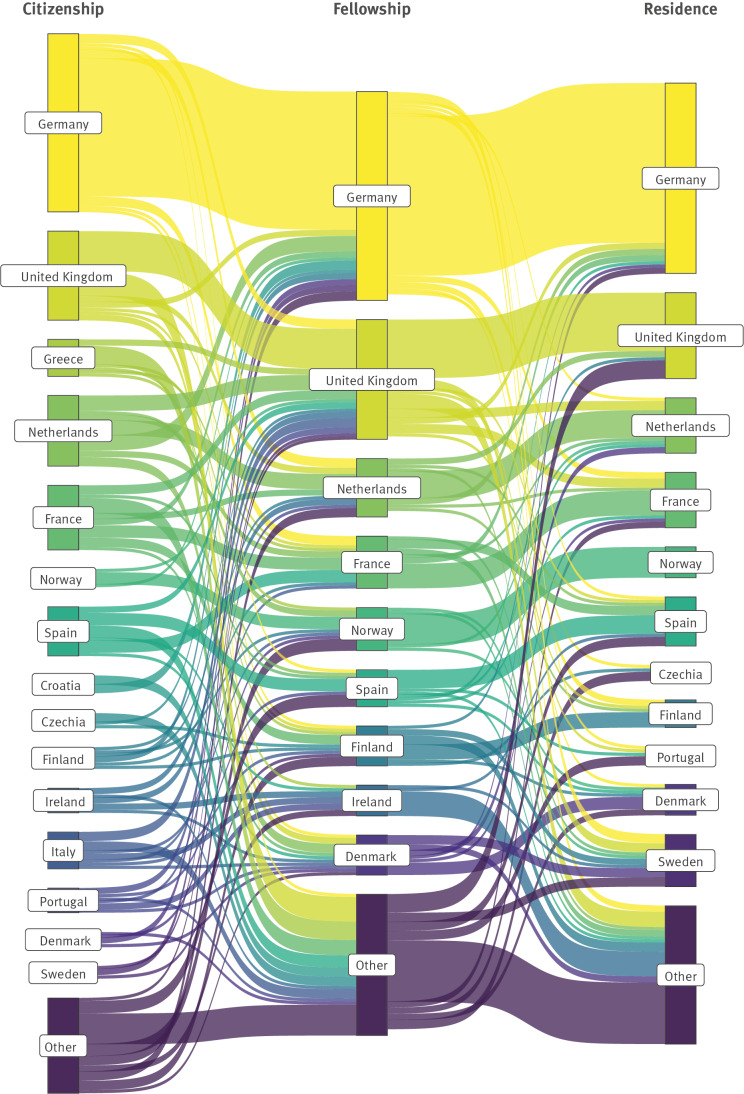
Sankey diagram showing the movement between country of citizenship, fellowship and (current) residence of participants of the EPIET Alumni Network, Member Survey, October‒December 2021 (n = 281)

The majority of respondents had acquired their first job after the fellowship in their country of citizenship (45%) or the country of fellowship (29%). The first job after the fellowship was more often in the country of citizenship among ECDC MS-track alumni (77%) than in ECDC EU-track alumni (38%). An overview of the movements of survey respondents through countries of citizenship, fellowship and current (2021) residence is shown in Figure 1. Supplementary Figure S1 includes the corresponding maps with the number of respondents per country of citizenship, fellowship and 2021 residence.

EPIET: European Programme for Intervention Epidemiology Training.

Countries for which fewer than five respondents indicated citizenship are grouped as ‘other’.

Member State-track alumni had a higher proportion of first jobs in their country of citizenship, which can be explained by the fact that most MS-track fellows already held a position within the public health sector before the fellowship and, after graduating, returned to the same job with additional skills.

Even though our survey captures data from alumni from many different countries, it may not be representative of the countries of origin, training and current work location of all EAN members. Nonetheless, to achieve and maintain an effective response to infectious disease threats throughout Europe, it is important that field epidemiologists and public health microbiologists are trained and retained equally in all European countries.

### Evolving skills and competencies

**Figure 2:**
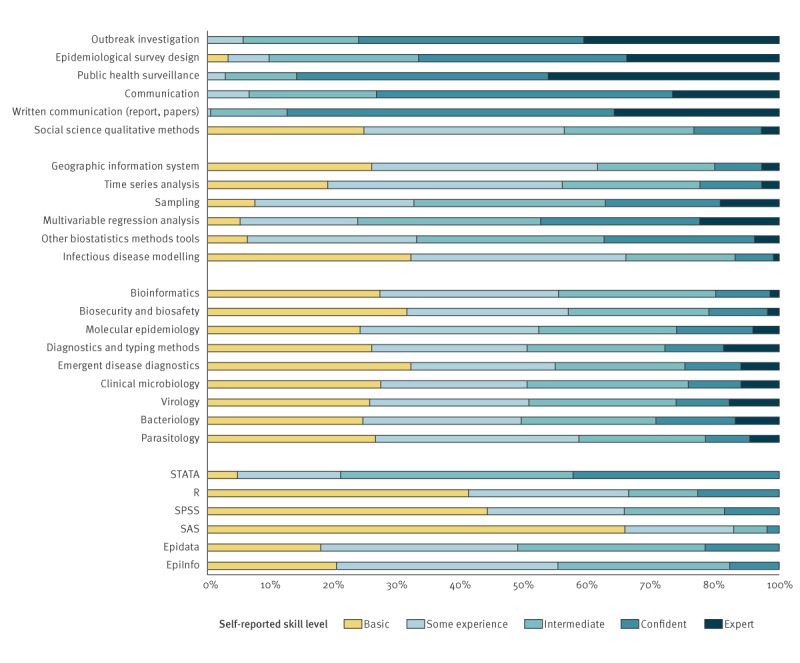
Competences and technical skills of respondents to the EPIET Alumni Network, Member Survey, October–December 2021 (n = 195)

A majority (89%, 250/281) of participants already had a medical, veterinarian, and/or PhD degree when they started their fellowship. Of the 192 European fellowship alumni respondents, 173 (90%) indicated that the skills and competencies they acquired during their fellowship helped them to perform better in their role compared their abilities before the fellowship. The self-reported level of expertise in a range of competencies and technical skills are shown in Figure 2. Perceived skills in core competencies in field epidemiology and public health microbiology were high, e.g. outbreak investigations, epidemiological surveys, public health surveillance. Competencies in epidemiology and data analysis were scored higher among EPIET-respondents than EUPHEM-respondents. See Supplementary Figures S2-A and B for mean scores on the self-reported competencies.

EPIET: European Programme for Intervention Epidemiology Training.

The answer categories were: basic knowledge/no practical experience, some/limited experience, intermediate confidence in practice, confident (independent professional) and expert (can teach).

Competence in the statistical software package R [12] was highest among most recently graduated respondents. R is a tool that has become increasingly used for epidemiological analyses in field epidemiology; its general uptake in use has become more global in recent years and it has been implemented in the fellowship programme [13]. Supplementary Figure S2-C gives an overview of self-reported competence on statistical and data analysis software packages (showing a mean score, on a scale from 0 (low) to 5 (high)); the skill level for EpiInfo [14] and EpiData [15] has declined among the more recent graduates. Competencies and technical skills related to public health microbiology showed an overall score that is relatively low (Figure 2) but much higher among EUPHEM-respondents, which is coherent with the background of these alumni and aim of the EUPHEM training programme. The skills and competencies of the EUPHEM trainees can be found in Supplementary Figure S2-D.

### Contribution to the COVID-19 response

European EPIET and EUPHEM alumni were heavily involved in the COVID-19 pandemic response at (sub)national and international levels, many of whom are middle/senior-level professionals. In total, 215 of 281 survey participants were involved in the COVID-19 response, mostly using skills and competencies related to surveillance (80%; n = 171), communication (67%; n = 144), outbreak investigations (48%; n = 103) and management (47%; n = 101).

Reported skills and competencies reported by 153 participants that could benefit from further strengthening were centred around data management/analysis (24%; n = 36), communication (21%; n = 32), mathematical modelling (11%; n = 17) and leadership/team management (10%; n = 16).

Our survey results show that communication, leadership and management skills were frequently needed during the pandemic, but that participants felt these skills were often lacking. These universally applicable competencies, i.e. leadership and communication, could be accommodated through adaptation of ECDC fellowship curriculum and investment in post-fellowship training. This is paramount to maintaining a contemporary, responsive workforce and fostering public health leaders of the future. Other research also has shown that, to ensure rapid and effective response to acute public health emergencies, the health security workforce needs skills such as leadership, communication, interpersonal skills and specialist training in emergency response, and that this is inadequate in current training models globally [16].

### Benefits and expectations from EPIET Alumni Network membership

**Figure 3:**
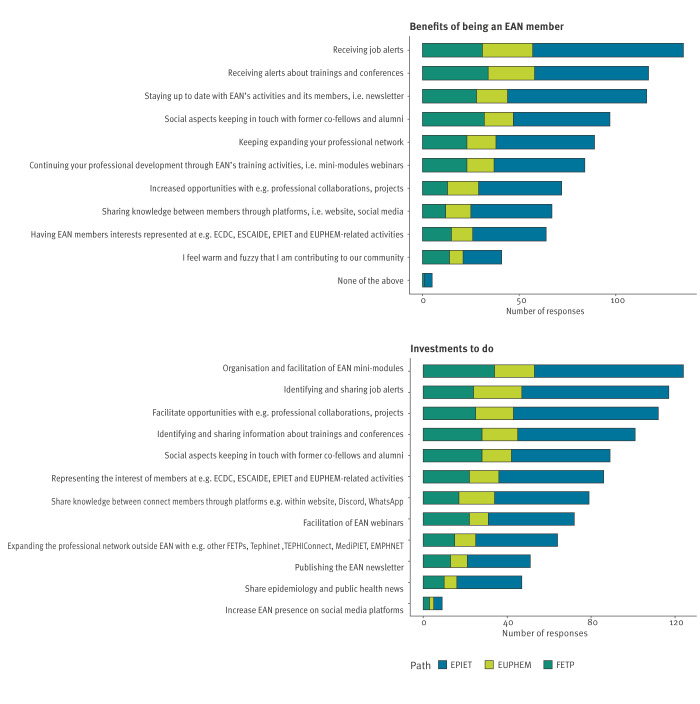
Benefits of EPIET Alumni Network membership, Member Survey, October‒December 2021

EAN: EPIET Alumni Network; ECDC: European Centre for Disease Prevention and Control; EMPHNET: Eastern Mediterranean Public Health Network; EPIET: European Programme for Intervention Epidemiology Training; ESCAIDE: European Scientific Conference on Applied Infectious Disease Epidemiology; EUPHEM: European Public Health Microbiology Training Programme; FETP: Field Epidemiology Training Program; Tephinet: Training Programs in Epidemiology and Public Health Interventions Network; TEPHIConnect: Tephinet’s online and mobile networking platform.

Career development, job opportunities and social networking were among the most frequently cited benefits and expectations of EAN membership (Figure 3). Participants responded that they would like to receive additional training (in scientific modules or webinars) – among others – on the following topics: humanitarian emergencies, communication with media and the public, funding opportunities, geographic information systems (GIS), time series analysis and ‘laboratory for epidemiologists’. Respondents would like the EAN to continue investing time in the organisation of scientific modules, identifying and sharing job alerts, facilitating opportunities for professional collaboration on projects, and identifying and sharing information on training opportunities.

Following the 2021 survey suggestions, EAN has organised a mini-module on GIS, a pre-European Scientific Conference on Applied Infectious Disease Epidemiology (ESCAIDE) mini-module on media communication and infodemic management, a training on R, and several webinars including one on zoonosis and one on mobile laboratory implementation in low resource settings. In addition, the EAN is currently preparing a mini-module on molecular epidemiology and a Go.Data training in collaboration with the Global Outbreak and Response Network (GOARN).

## Present and future field epidemiology and public health microbiology workforce 

Together, we look back on more than 25 years of training of a European workforce of field epidemiologists and 15 years of training public health microbiologists. The EPIET and EUPHEM fellowships contribute to a strong foundation of public health professionals that are connected across Europe and globally. During the 25-year celebration of the EPIET programme in November 2022, the past, the present, and the future of field epidemiology [17] was discussed among the EAN members who attended this event. The strength of the EAN lies also in operating beyond politically restricted and geographical boundaries, both within Europe and beyond, as EAN also includes members outside Europe. One of the future challenges for the EAN it to find the right balance between keeping strong ties between European fellowship alumni (which is its primary goal and is easier with a smaller network) and being enriched by public health professionals from around the world that can enter as external members and provide ties with other FETP-networks. 

This perspective underlines the importance of ensuring an agile, responsive network of highly skilled public health professionals. A strong network benefits individual professionals, but also fosters collaboration across Europe, as was demonstrated during response to the COVID-19 pandemic. However, coordinating and maintaining the EAN network is only possible through the substantial time invested by the EAN Advisory Board members and the invaluable support of EAN members. It is important that network members proactively engage with the network by investing ‘time, place and person’.

The contribution made by field epidemiologists and public health microbiologists to improving public health in Europe is enhanced through membership of the expanding EAN. The need for sustained investment in competency and skills development remains, and this should be monitored over time as needs change [18]. The distinctive and fundamental parts of field epidemiology should be cherished and safeguarded [19]. 

## Conclusions 

During the COVID-19 pandemic, the utility and usefulness of field epidemiology and public health microbiology programme graduates was demonstrated. This clearly underlines the value of the ECDC Fellowship training programme and affiliated programmes. The culture of collaboration across Europe and training of the next generation of competent professionals can be optimised through continued investment in these programmes. Ensuring sufficient funding, and even considering expansion in funding and available fellowships per cohort, will remain crucial to maintaining and increasing the professional and well-trained workforce that is needed for optimal response to infectious diseases and protection of public health [20]. Further strengthening of (sub)national public health systems in the European region and thereby also creating opportunities for a well-trained workforce is the key for a healthier Europe in the future.

## Supplementary Data

496000 Supplement
