# Supplementary material for: Field Epidemiology and Public Health Microbiology training: capturing the alumni perspectives of the training’s impact
Source: Euro Surveill. 2023 Sep 7;28(36):2300388. doi: 10.2807/1560-7917.ES.2023.28.36.2300388 (PMC10486191; doi:10.2807/1560-7917.ES.2023.28.36.2300388)

Supplementary materials.

"This supplementary material is hosted by Eurosurveillance as supporting information alongside the article *Field Epidemiology and Public Health Microbiology training: capturing the alumni perspectives of the training's impact* on behalf of the authors who remain responsible for the accuracy and appropriateness of the content. The same standards for ethics, copyright, attributions and permissions as for the article apply. Eurosurveillance is not responsible for the maintenance of any links or email addresses provided therein."

Supplementary Figure S1.
Maps showing the number of respondents per country of citizenship, fellowship and 2021-residence (N=246).

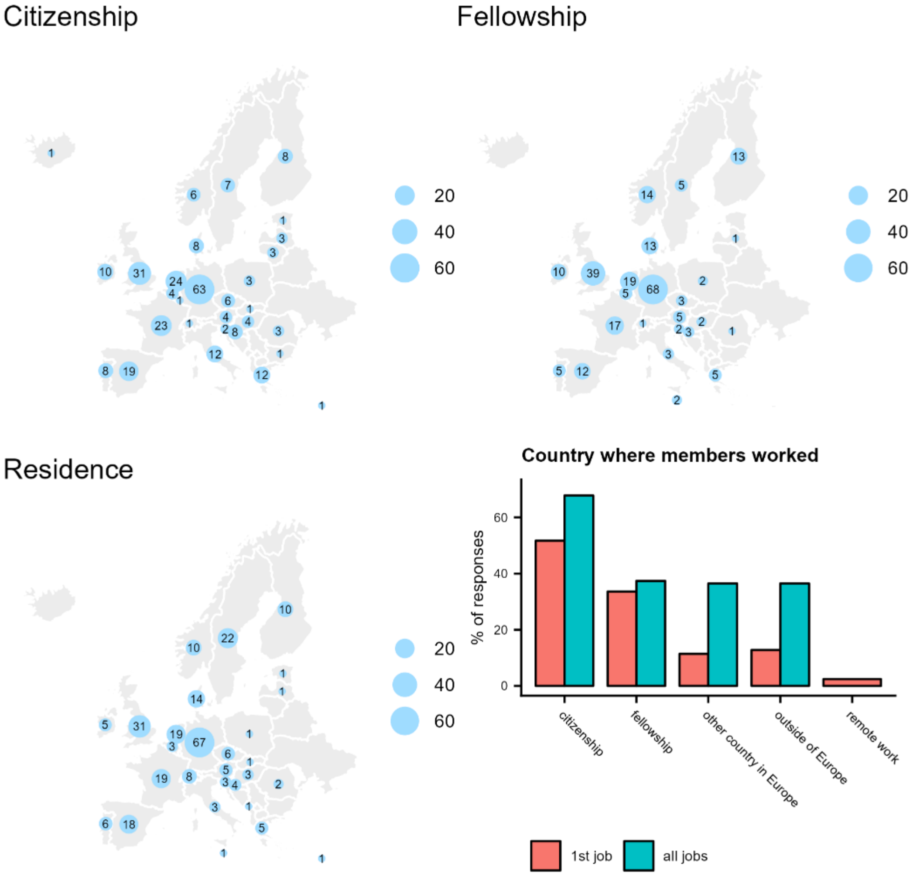

Supplement Figure S2.
Mean scores (scale 1-5) on the self-reported competencies (A and B), software skills (C), and technical and microbiological skills (D) stratified by fellowship track (EPIET, EUPHEM or FETP) and year of starting the fellowship (N=195).

Legend for panel A, B, C and D: the years of starting the fellowship are grouped as fellows: 1995-1999, 2000-2004, 2005-2009, 2010-2014, 2015-2019. Mean scores were calculated for the answers with the following labels: 1. Basic knowledge/No practical experience, 2. Some/ limited experience, 3. Intermediate confidence in practice, 4. Confident (Independent professional), 5. Expert-can teach.

Figure S2-A: Mean scores (1-5) for key competencies in field epidemiology and public health microbiology

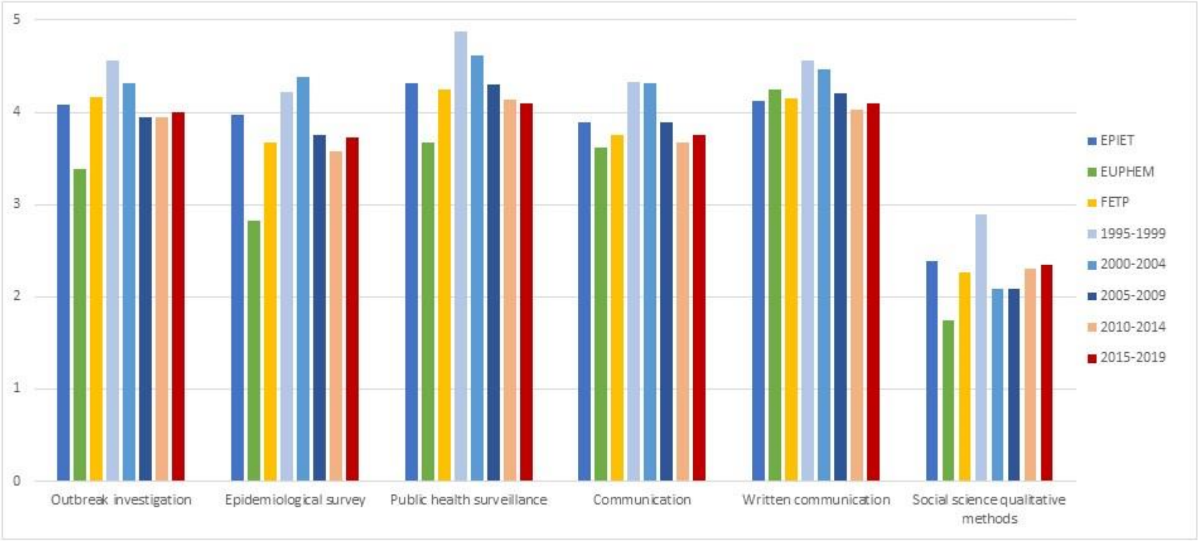

Figure S2-B: Mean scores (1-5) for competencies in epidemiology and data analysis

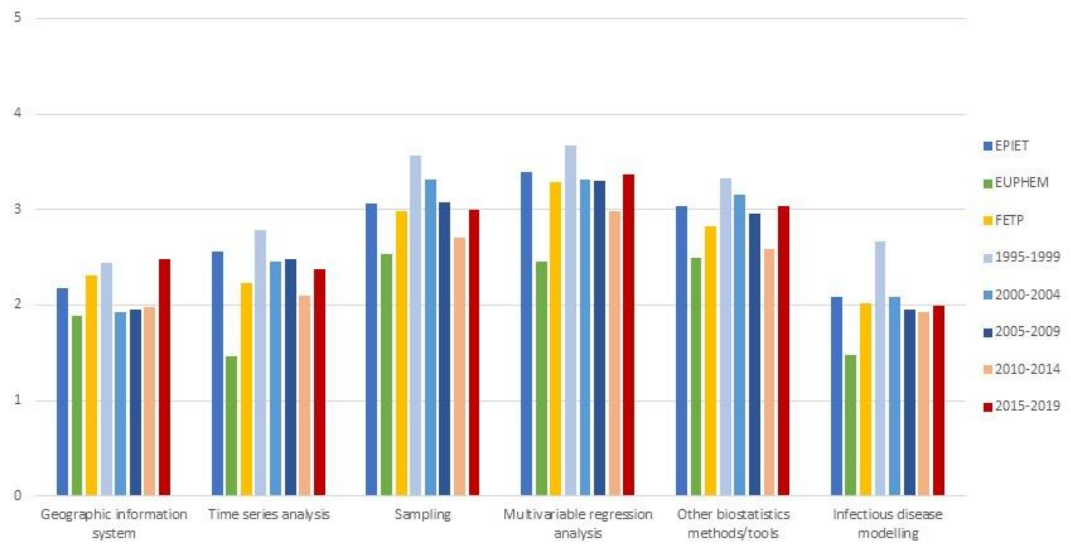

Figure S2-C: Mean scores (1-5) for competencies in statistical software packages

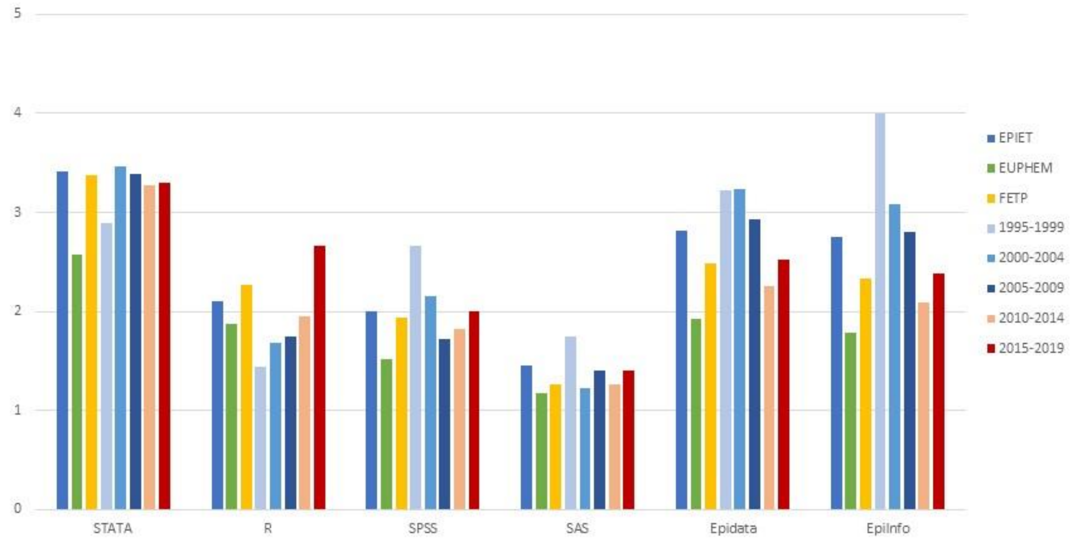

Figure S2-D: Mean scores (1-5) for microbiological and technical skills and competencies

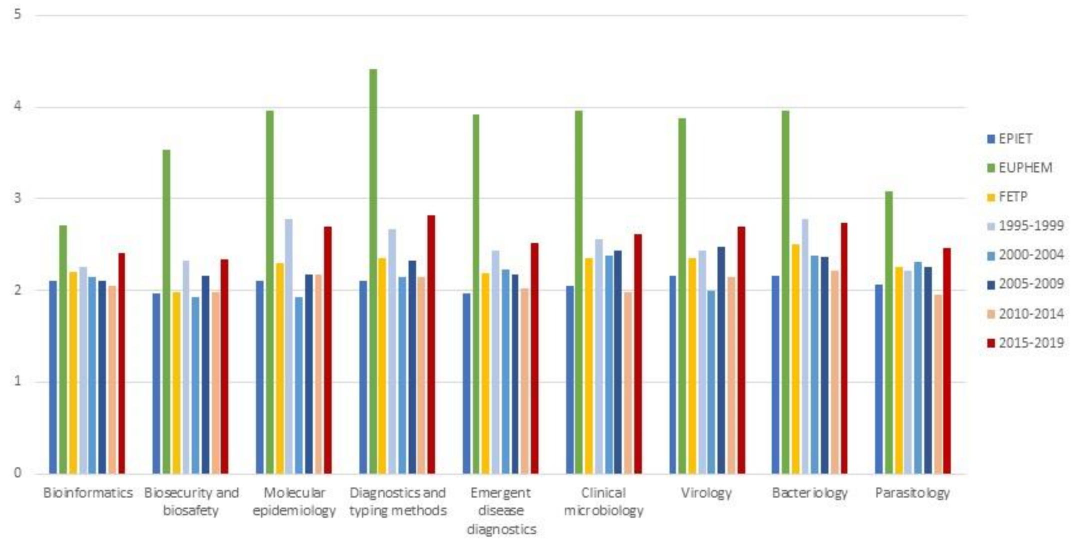

Supplement: Supplement [file 23-00388_TOSTMANN_Supplement.pdf]
